# Forage-free diet in finishing cattle: Effects on performance, ruminal fermentation, and enteric methane mitigation

**DOI:** 10.1016/j.vas.2026.100608

**Published:** 2026-02-28

**Authors:** Vagner Ovani, Leandro Sâmia Lopes, Lumena Souza Takahashi, Patricia Spoto Corrêa, Patricia Righeto, Theyson Duarte Maranhão, Thaynã Gonçalves Timm, Helder Louvandini, Adibe Luiz Abdalla

**Affiliations:** a Animal Nutrition Laboratory, Centre for Nuclear Energy in Agriculture, University of São Paulo, Piracicaba, Sao Paulo, CEP 13400-970, Brazil; b Animal Science Department, Faculty of Animal Science and Veterinary Medicine; Federal University of Lavras, Lavras, Minas Gerais, CEP 37.200-000, Brazil; c Center for Carbon Research in Tropical Agriculture (CCARBON) - University of São Paulo, Piracicaba, São Paulo, Brazil

**Keywords:** High-concentrate feeding, Plant secondary metabolites, Acidosis risk, Rumen health, Microbial adaptation, Feed efficiency, *In vitro* gas production, Feedlot

## Abstract

Ruminant production is largely pasture-based, and although this system is beneficial during the cow-calf and growing phases, it often becomes nutritionally limiting during finishing, delaying slaughter and increasing greenhouse gas emissions. High-grain diets accelerate weight gain and may reduce enteric methane (CH_4_) emissions per unit of animal output, but they increase the risk of ruminal acidosis. Ionophores are effective in mitigating this disorder, yet their use is restricted in several countries, stimulating interest in sustainable alternatives such as plant-derived bioactive compounds. This study evaluated the effects of low-grain, high-grain, and forage-free diets supplemented with a functional additive (LM+), a blend of rumen-modulating secondary metabolites, on animal performance and ruminal fermentation under *in vivo* finishing conditions. Forty-five crossbred Girolando cattle were assigned to three diets for 147 days. Animal performance, ruminal pH, short-chain fatty acids, lactate concentration, and selected microbial groups were assessed *in vivo*. In addition, an *in vitro* gas production assay was conducted at the end of the *in vivo* trial, using ruminal fluid from adapted animals, to provide complementary mechanistic information on organic matter degradability and fermentation partitioning. The forage-free diet resulted in the highest average daily gain (1.43 kg day^-1^) compared to low-grain (0.49 kg day^-1^) and high-grain diets (1.26 kg day^-1^; *P* < 0.001). Increasing grain inclusion reduced ruminal pH (lowest in the forage-free diet: 5.59; *P* < 0.001) and increased short-chain fatty acid and lactate concentrations (*P* < 0.001). Quantitative PCR showed decreases in fibrolytic bacteria and methanogenic archaea with increasing grain levels (*P* < 0.01). Principal component analysis indicated clear dietary separation, associating the forage-free diet with greater final body weight and distinct ruminal fermentation patterns. These findings indicate that forage-free diets supplemented with plant-derived additives improved cattle performance and altered ruminal fermentation under finishing conditions.

## Highlights

• Forage-free diet with plant-based additive improved feed intake and growth in finishing cattle.
• Ruminal health was maintained despite lower pH and microbiota shifts.
• Forage-free diet was associated with reduced estimated methane intensity.

## 1 Introduction

Approximately 90 % of the Brazilian cattle herd is raised under grazing systems (Oliveira Silva et al., 2017), occupying about 173 million hectares of pastureland, which accounts for around 72 % of the country’s arable land (FAOSTAT, 2025). This scenario is justified by the fact that pasture remains the most cost-effective feed source for ruminants (Bargo et al., 2003), especially during the cow-calf and growing phases. However, in the finishing phase, exclusive reliance on pasture can extend the time to slaughter, since it often fails to meet the nutritional requirements of animals. Forages were not designed to provide a balanced nutrient profile for optimal performance (Wilkinson & Lee, 2018), particularly due to fluctuations in yield and quality during the dry season (Silva et al., 2021), which lead to inconsistent weight gain. Moreover, low-quality forage, especially with poorly digestible fiber, modifies ruminal fermentation patterns, leading to increased methane (CH_4_) production and reduced dietary energy efficiency (Hua et al., 2022). Consequently, a longer fattening period combined with higher CH_4_ emissions per unit of feed consumed makes this production practice less sustainable from both environmental and economic perspectives.

One strategy to overcome these challenges is the inclusion of grains in the finishing diets of cattle. High-grain diets increase daily energy intake, provide a greater supply of essential amino acids, and improve the ruminal balance between fermentable energy and degradable protein (Wilkinson & Lee, 2018). Thus, grain-rich feedlot diets maximize weight gain in shorter periods, improving production efficiency and profitability (Castillo et al., 2004; Hernández et al., 2014), thereby reducing CH_4_ emissions per unit of meat produced, both due to the faster growth rate and shorter time to market, and through modulation of ruminal fermentation, particularly by decreasing the acetate proportion and concomitantly increasing the propionate proportion (Beauchemin & McGinn, 2005).

Despite these economic and environmental benefits of high-grain diets during the finishing phase, providing maximum grain inclusion without causing ruminal acidosis remains a major challenge. Diets with little or no roughage disturb the ruminal microbiome, predispose animals to acidosis, and compromise health and performance. Digestive disorders such as ruminal acidosis rank second only to respiratory diseases as the main causes of reduced animal performance and production efficiency (Castillo et al., 2004; Nagaraja & Lechtenberg, 2007; Plaizier et al., 2012).

To address this challenge, feeding strategies and the use of fermentation-modulating additives have been evaluated as alternatives to enable high-grain diets without causing acidosis. Management approaches include increasing dietary fiber content, controlling the type and processing of grains to reduce the rate of starch fermentation, and avoiding abrupt drops in ruminal pH. Supplementation with buffers can neutralize acids in the rumen, while organic acids, plant-based products, and probiotics stimulate lactate utilization, stabilize ruminal pH, and maintain rumen health in high-concentrate diets (Hernández et al., 2014). Among additives, ionophores (*e.g.*, monensin, salinomycin, lasalocid, narasin, maduramycin, semduramicin, and laidlomycin propionate) stand out for modifying rumen fermentation, increasing feed efficiency, and selectively targeting Gram-positive bacteria, thereby reducing lactate production and serving as an effective strategy to prevent acidosis disorders in high-grain diets (Prusty et al., 2024; Seema et al., 2025).

Restrictions or bans on conventional additives in several countries have driven the search for more sustainable alternatives, such as plant extracts and bioactive compounds, to control rumen fermentation, mitigate CH_4_ production, and maintain productive efficiency (Castillo et al., 2004; Patra & Saxena, 2011). In this context, plant-derived bioactive compounds have emerged as promising modulators of ruminal microbiota and fermentation. Although studies are still limited, several classes of phytobiotics (*e.g.*, herbs, botanicals, essential oils, and oleoresins) have shown the ability to influence rumen microbial populations. Carefully combining these metabolites may allow the manipulation of rumen microbial fermentation while reducing metabolic disorders such as acidosis (Hernández et al., 2014).

In forage-free diets, rapid starch fermentation increases short-chain fatty acid and hydrogen production, creating conditions that favor ruminal acidification and methane formation. Essential oil–based additives have been proposed as alternatives to ionophores due to their ability to selectively modulate ruminal microbial populations, particularly Gram-positive bacteria, through interactions with cell membrane integrity. This modulation alters fermentation patterns, reduces the acetate:propionate ratio, and improves energetic efficiency, potentially decreasing methane emissions while stabilizing ruminal fermentation under high-grain feeding conditions (Benchaar et al., 2008; Burt, 2004; Calsamiglia et al., 2007).

The combined evaluation of animal performance, fermentation end-products, specific ruminal microbial groups, and complementary *in vitro* variables can provide a multidimensional assessment of the dietary effects on rumen function. Therefore, this study aims to evaluate low and high-grain diets compared to a forage-free diet supplemented with a functional additive (LM+), composed of a blend of rumen-modulating essential oils and short-chain fatty acids (SCFA), by assessing their effects on ruminal fermentation parameters, *in vitro* gas production, CH_4_ emissions, ruminal microbiota composition, and their effectiveness in improving animal performance during long-term *in vivo* trials. We hypothesize that the inclusion of this additive may stabilize ruminal pH, reduce lactic acid accumulation, lower CH_4_ emissions, and enhance animal performance, supporting its use as a sustainable strategy for acidosis control in forage-free diets.

## 2 Material and methods

### 2.1 Experimental site

The *in vivo* assay was carried out at the feedlot of the Training and Capacity-Building Center, located in Martinho Campos, Minas Gerais, Brazil. The *in vitro* assay to evaluate ruminal fermentation parameters of the experimental diets was conducted at the end of the *in vivo* assay at the Animal Nutrition Laboratory (LANA) of the Center for Nuclear Energy in Agriculture (CENA), University of São Paulo (USP) (Piracicaba, São Paulo, Brazil), using ruminal contents from 24 animals from the *in vivo* assay, following animal use ethics committee protocol No 16/2018*.*

### 2.2 *In vivo* assay

In the *in vivo* assay, animals were stratified by initial body weight and then randomly assigned to three treatments, with 15 animals per treatment (*n* = 45), and housed in collective pens equipped with waterers and feeders that allowed all animals to feed simultaneously. Entire crossbred Girolando male cattle (3/4 to 7/8 Holstein × Gir) were used. The animals had an average initial body weight (IBW) of 239 ± 5.3 kg and an average age of 7 ± 1.8 months. All animals originated from the same herd and were managed under the same routine conditions prior to the experiment. An antiparasitic treatment was administered before feedlot entry.

Three experimental diets (Table 1) were used: a low-grain diet (90 % forage and 10 % concentrate); a high-grain diet (70 % concentrate and 30 % forage), with concentrate composed of corn, soybean meal, cottonseed, urea, and a mineral premix, and the forage consisting of ground *Brachiaria* spp. hay to prevent feed selection; and a forage-free diet (85 % whole corn grain and 15 % protein-vitamin-mineral pellet, without forage inclusion). The pellet (15 % of diet dry matter) was composed of rice bran, cottonseed meal, soybean meal, corn gluten meal, macro and microminerals, and a functional additive (Factor LM+®) (Lopes et al., 2023). Factor LM+® is a proprietary blend of rumen-modulating compounds, including essential oils derived from garlic (*Allium sativum*), eucalyptus (*Eucalyptus spp*.), oregano (*Origanum vulgare*), neem (*Azadirachta indica*), yucca (*Yucca schidigera*), and annatto (*Bixa orellana*). In addition, the additive contains short-chain fatty acids (propionic, fumaric, malic, and ascorbic acids), tannins, and nucleotides.

**Table 1:** Composition and chemical analysis of the experimental diets offered during the *in vivo* assay.

Ingredients	Experimental diets ( %)		
	Low-grain	High-grain	Forage-free
Forage^1^	90	30	-
Protein-mineral pellet^2^	-	-	15
Concentrate^3^	10	70	85
Chemical composition (g kg^-1^ DM)			
Neutral detergent fiber	693	376	124
Acid detergent fiber	361	243	58
Lignin	100	58	28
Crude protein	95	191	149
Ash	94	73	58
Ether extract	34	38	36

1 *Brachiaria* spp hay;.

2 Protein-vitamin-mineral pellet including Factor LM+, a proprietary additive based on essential oils and SCFA with rumen-modulating properties;.

3 The concentrate was composed of 48.0 % ground corn, 25.4 % soybean meal, 20.0 % cottonseed, 0.9 % urea, and 5.7 % mineral premix.

The experimental period lasted 147 days of confinement. Animals were fed three times daily (7:00, 11:00, and 16:00 h), and the amount of feed was adjusted to maintain 5–10 % refusals. Body weight was recorded every 21 days using an electronic scale, after a 12-hour solid feed withdrawal, to monitor average daily gain (ADG). ADG was calculated as the difference between final and initial body weight, divided by the number of confinement days. Dry matter intake (DMI) was measured daily at the pen level as the difference between feed offered and refusals collected before the first feeding of the day. Individual DMI was estimated by proportionally allocating total pen intake based on individual body weight. Neutral detergent fiber intake (NDFI) and total digestible nutrient intake (TDNI) were calculated from DMI and the chemical composition of each experimental diet. Enteric CH_4_ emissions were estimated using a single prediction equation across all dietary treatments to maintain methodological consistency when comparing low-grain, high-grain, and forage-free diets. The equation of Lingen et al. (2019) was used: CH_4_ *(g day^-1^) = 17.9 + 0.732 × For + 0.226 × BW*, where *BW* is body weight (kg) and *For* is the dietary forage proportion ( % of diet DM). This model was selected based on its highest predictive performance among several evaluated equations, with body weight identified as the variable explaining the largest proportion of variance in the sensitivity analysis reported by Monteiro et al. (2024). In addition, the model incorporates dietary forage proportion, a variable that is relevant for distinguishing the contrasting diets evaluated in this study.

At the end of the experimental period, ruminal fluid was collected from all animals four hours after the morning feeding using an esophageal probe. Ruminal pH was measured on-site using a calibrated Akso pH meter, previously standardized with buffer solutions at pH 4, 7, and 10 according to the manufacturer’s instructions. For each sample, 125 *g* of ruminal fluid were immediately frozen and sent to CBOLAB Laboratory in Valinhos, São Paulo, Brazil, for analysis of SCFA and lactate.

### 2.3 *In vitro* assay

At the end of the *in vivo* assay, ruminal contents were collected from 24 cattle (8 per diet) using an esophageal probe, immediately transferred to CO_2_-flushed thermal flasks to maintain anaerobic conditions and temperature, and transported to the LANA-CENA-USP for the *in vitro* assay. Upon arrival, approximately 10 mL of ruminal content from each of the 24 animals were immediately sampled and stored in an ultra-freezer (−80 °C) for subsequent DNA extraction, to quantify total Bacteria, Archaea, Fungi, Protozoa, *Fibrobacter succinogenes, Ruminococcus albus, Ruminococcus flavefaciens*, and mcrA (methyl-coenzyme M reductase, a methanogenic marker) gene (Corrêa et al., 2021). The remaining ruminal fluid was maintained in an *in vitro* incubator (TE-150, Tecnal, São Paulo, Brazil) (39 °C, 25 rpm) until the start of the *in vitro* gas production technique, approximately 24 h after collection.

The *in vitro* gas production technique followed Theodorou et al. (1994), with adaptations from Mauricio et al. (1999) and Bueno et al. (2005). For each thermal flask (*n* = 8 per diet), triplicate samples of each experimental diet (representing the diets provided during the *in vivo* assay) were prepared, totaling 72 fermentation substrates. Specifically, 1 g of each test diet was placed in Ankom F57 filter bags and transferred to 160 mL glass bottles, along with 50 mL of incubation medium and 25 mL of ruminal content from the animals fed each respective diet. The incubation medium was prepared with the solutions described by Menke et al. (1979).

The bottles were sealed with rubber stoppers and incubated in a forced-air oven (MA 035; Marconi, São Paulo, Brazil) at 39 °C for 24 h. Gas pressure (psi) was measured at 2, 4, 8, 12, 16, and 24 h to determine the volume of gas produced, and gas samples were collected at each time for CH_4_ analysis. The inside pressure (psi) was measured with a semi-automatic system using a pressure transducer and a datalogger (Bizzuti et al., 2023; Bueno et al., 2005; Mauricio et al., 1999; Pérez-Márquez et al., 2023), and was converted to gas volume according to Ovani et al. (2025). CH_4_ concentration was determined by gas chromatograph (GC-2010, Shimadzu, Tokyo, Japan) following Lima et al. (2018).

At the end of the incubation period, the residue in each bag was washed with neutral detergent solution to determine truly degraded organic matter, expressed as *in vitro* degradability of organic matter (IVDOM) (Sakita et al., 2020). Approximately 20 mL of incubation fluid per bottle were collected for pH measurement and stored frozen for SCFA analysis (Lima et al., 2018). Organic matter (OM) from each incubated substrate was partitioned into degraded and undegraded fractions, gas losses (*i.e.*, CO_2_, CH_4_, and water), OM used for SCFA production, and OM used for microbial biomass synthesis, according to the stoichiometric considerations proposed by Blümmel et al. (1997).

### 2.4 Statistical analysis

All statistical analyses were performed using R software version 4.3.2 (R Core Team, 2023) and the packages dplyr (Wickham et al., 2021), lme4 (Bates et al., 2015), emmeans (Lenth, 2021), and ggplot2 (Wickham, 2016). The experimental design was a completely randomized design with three dietary treatments (low-grain, high-grain, and forage-free).

For *in vivo* and *in vitro* variables, normality (Shapiro-Wilk) and homoscedasticity (Levene's tests) were verified. When assumptions were met, analysis of variance (ANOVA) was applied, and treatment means were compared using Tukey’s honestly significant difference (HSD) test with a significance level of *P* < 0.05.

Principal component analysis (PCA) was performed using the FactoMineR (Lê et al., 2008) and factoextra (Kassambara & Mundt, 2020) packages, including selected performance, fermentation, partitioning, and microbiota variables. Data were centered, scaled, and missing values imputed via imputePCA() function from the missMDA (Josse & Husson, 2016) package. Treatments were overlaid on the PCA biplot using confidence ellipses to visualize clustering.

## 3 Results

### 3.1 *In vivo* performance and CH_4_ emissions

Initial body weight (IBW) did not differ among treatments (*P* = 0.357). However, final body weight (FBW), ADG, and DMI were significantly influenced by the diets (*P* < 0.001), with higher values observed in animals fed the high-grain and forage-free diets, respectively (Table 2). Diet affected CH_4_ emissions (g day^-1^ and g kg^-1^ DMI; *P* < 0.001), which decreased progressively with increasing grain inclusion, reaching the lowest values in the forage-free diet (Table 2).

**Table 2:** Performance, intake, CH_4_ emissions, and *in vivo* ruminal parameters of confined cattle fed diets with different energy levels.

Variables	Diets			SE	P value
	Low-grain	High-grain	Forage-free		
IBW (kg)	228.0	235.1	231.4	3.43	0.357
FBW (kg)	340.4^b^	434.5^a^	459.6^a^	14.01	<0.001
ADG (kg animal^-1^ day^-1^)	0.49^b^	1.26^a^	1.43^a^	0.068	<0.001
DMI (kg day^-1^)	9.04^b^	11.00^a^	11.44^a^	0.264	<0.001
NDFI (kg day^-1^)	5.20^a^	2.6^b^	1.19^c^	0.0721	<0.001
TDNI (kg day^-1^)	3.85^c^	4.60^b^	5.73^a^	0.1181	<0.001
Ruminal pH	6.73^a^	6.29^a^	5.59^b^	0.141	<0.001
Total SCFA (mmol l^-1^)	63.3^c^	109.0^b^	155.2^a^	11.42	<0.001
A:P ratio	4.23^b^	3.50^b^	1.18^a^	0.274	<0.001
Lactate (mmol l^-1^)	1.20^a^	5.03^b^	13.96^c^	0.760	<0.001
CH_4_ (g day^-1^)	161^a^	138^b^	122^c^	3.17	<0.001
CH_4_ (g kg^-1^ DMI)	17.8^c^	12.6^b^	10.6^a^	0.082	<0.001

IBW, initial body weight; FBW, final body weight; ADG, average daily gain; DMI, dry matter intake; NDFI, neutral detergent fiber intake; TDNI, total digestible nutrients intake; SCFA, short-chain fatty acids; A:P ratio, acetate: propionate ratio; CH_4_, methane.

a,b,c Means followed by different superscript letters within a row indicate significant differences among dietary treatments according to Tukey’s test (*P* < 0.05).

Ruminal pH differed among treatments (*P* < 0.001), with lower values observed as dietary energy increased. The concentration of total SCFA was also affected by the diets (*P* < 0.001), with higher values associated with higher energy diets. The acetate:propionate (A:P) ratio and lactate concentration were significantly affected by the diets (*P* < 0.001). The lowest A:P ratio and the highest lactate concentration were observed in animals fed the forage-free diet.

Ruminal microbiota composition, expressed as log of copy number per 10 ng of DNA, is presented in Table 3. Total bacterial abundance did not differ among treatments (*P* = 0.691). Fungal abundance was significantly affected by diet (*P* < 0.01), with lower values in the forage-free diet. Archaeal populations showed a tendency to differ among treatments (*P* = 0.050). Protozoal abundance varied significantly (*P* < 0.01), with the lowest values in the forage-free diet. Methanogen abundance (mcrA gene) differed among treatments (*P* = 0.017), with a reduction in the forage-free diet. *Fibrobacter succinogenes, Ruminococcus albus*, and *Ruminococcus flavefaciens* populations were all significantly affected by diet (*P* < 0.01), with lower abundances as grain levels increased. *Prevotella ruminocola* also differed among treatments (*P* = 0.015), with the lowest values detected in the forage-free diet.

**Table 3:** Ruminal microbiota composition (log of copy number/100 ng DNA) of cattle fed diets with different energy levels.

Variables	Diets			SE	P value
	Low-grain	High-grain	Forage-free		
Bacteria	8.34	8.28	8.34	0.064	0.691
Fungi	4.46^a^	4.11^a^	3.35^b^	0.143	<0.01
Archaea	5.16	4.72	4.78	0.130	0.050
Protozoa	7.00^a^	7.72^a^	5.10^b^	0.468	<0.01
mcrA (methanogens)	2.97^b^	2.73^a^^b^	2.42^a^	0.127	0.017
*Fibrobacter succinogenes*	5.24^a^	4.31^b^	2.90^c^	0.228	<0.01
*Ruminococcus albus*	5.87^a^	5.29^a^	4.20^b^	0.274	<0.01
*Ruminococcus flavefaciens*	4.62^a^	4.41^a^	3.55^b^	0.194	<0.01
*Prevotella ruminicolla*	5.42^a^	5.43^a^	4.40^b^	0.273	0.015

Values are expressed as log of copy number per 100 ng of DNA. SE, standard error.

a,b,c Means followed by different superscript letters within a row indicate significant differences among dietary treatments according to Tukey’s test (*P* < 0.05).

### 3.2 *In vitro* fermentation parameters

Gas production (GP) differed significantly among treatments after 24 h of incubation (*P* < 0.001), with greatest values in the forage-free diet (Table 4). CH_4_ production, expressed both per unit of incubated dry matter (CH_4_, mL g^-1^ DM) and per unit of degraded organic matter (CH_4_, mL g^-1^ DOM), also varied among treatments (*P* < 0.001 and *P* = 0.002, respectively), following the levels of grain inclusion.

**Table 4:** *In vitro* gas production parameters of cattle diets with different energy levels (24 h incubation).

Variables	Diets			SE	P value
	Low-grain	High-grain	Forage-free		
GP (mL g^-1^ DM)	35.8^a^	59.3^b^	89.5^c^	3.38	<0.001
CH_4_ (mL g^-1^ DM)	1.21^a^	2.19^a^	4.14^b^	0.259	<0.001
CH_4_ (mL g^-1^ DOM)	4.7^a^	5.5^a^	7.7^b^	0.33	0.002
IVDOM (g kg^-1^)	296^c^	456^b^	597^a^	23.4	<0.001
SCFA (mmol l^-1^)	68.7^b^	91.1^b^	178.6^a^	6.76	<0.001
A:P ratio	2.37^b^	2.03^b^	0.77^a^	0.094	<0.001

GP, gas production per dry matter incubated; CH_4,_ methane production; SCFA, short-chain fatty acids; IVDOM, *in vitro* degradability of organic matter; A:P ratio, acetate: propionate ratio; DM, dry matter; DOM, degradability of organic matter; SE: standard error.

a,b,c Means followed by different superscript letters within a row indicate significant differences among dietary treatments according to Tukey’s test (*P* < 0.05).

The IVDOM was significantly affected by the diets (*P* < 0.001), with higher degradability observed in treatments with increased grain levels. Similarly, the concentration of SCFA varied significantly (*P* < 0.001), with higher values detected in the forage-free diet.

The A:P ratio differed among treatments (*P* < 0.001), with the lowest value in the forage-free diet.

**Fig. 1:**
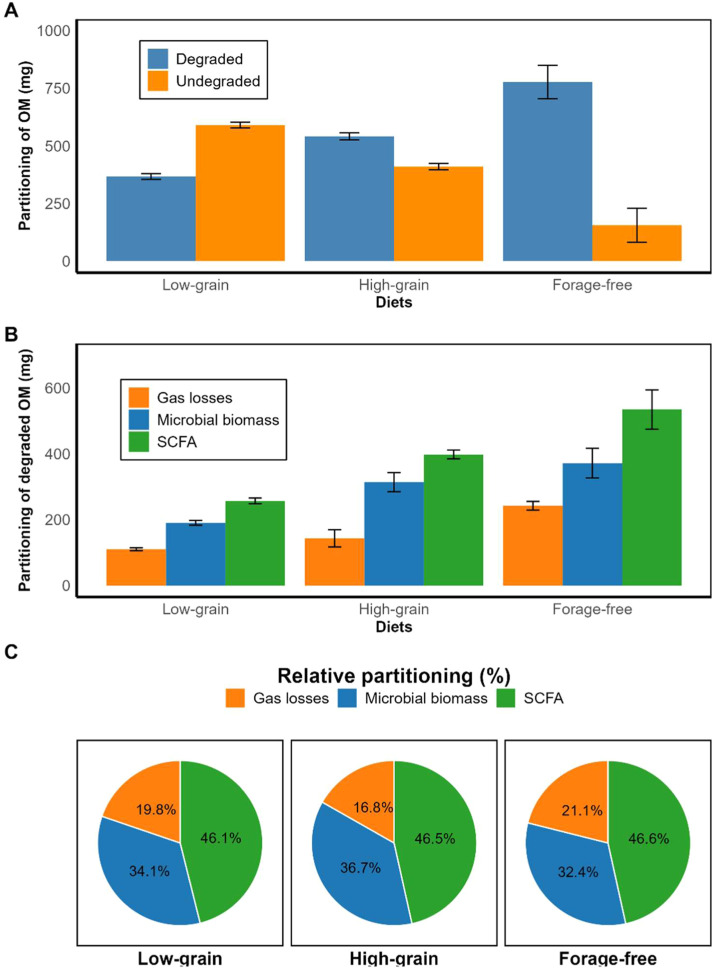
Partitioning of incubated organic matter (OM) (A), degraded OM (B), and relative partitioning (C) in cattle diets with different energy levels.

The partitioning of incubated and degraded organic matter is presented in Fig. 1. Degraded OM increased progressively with greater grain levels, while undegraded fractions decreased proportionally (Fig. 1A). Partitioning of degraded OM into gas losses, microbial biomass, and SCFA differed among diets (Fig. 1B). Higher microbial biomass and SCFA were associated with greater grain inclusion, whereas gas losses showed less variation. Relative partitioning of degraded OM is shown in Fig. 1C. Across all diets, SCFA represented the largest fraction, followed by microbial biomass and gas losses, with slight variations among treatments. The high-grain diet exhibited a greater proportion of OM directed to microbial biomass.

### 3.3 Principal component analysis (PCA)

**Fig. 2:**
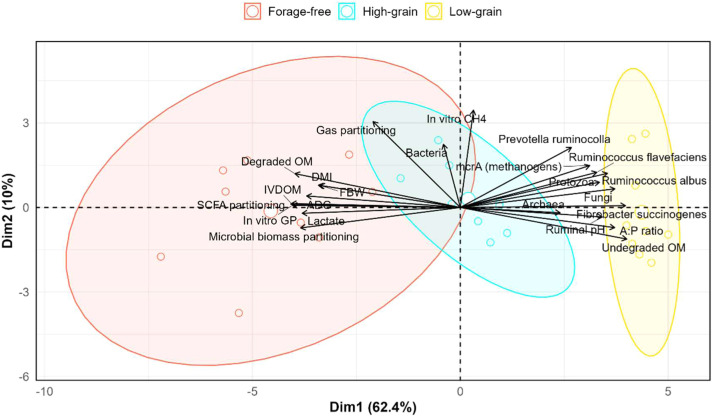
Principal component analysis (PCA) of *in vitro* fermentation parameters, microbial profile, and animal performance variables in confined cattle fed diets with different energy levels.

The PCA revealed distinct clustering of treatments according to dietary energy levels (Fig. 2). The first two principal components explained a substantial proportion of total variance, enabling clear separation among diets. Animals on the low-grain diet clustered on the right side, associated with greater abundance of *Fibrobacter succinogenes, Ruminococcus albus, Ruminococcus flavefaciens*, and *Prevotella ruminicola*, higher undegraded OM, and higher A:P ratio.

In contrast, animals on the forage-free diet clustered on the left associated with variables related to the extent and pattern of ruminal fermentation, including *in vitro* gas production, SCFA partitioning, lactate concentration, microbial biomass partitioning, and IVDOM. Additionally, performance variables (DMI, ADG, and FBW) were also correlated with this group.

The high-grain diet showed an intermediate distribution, partially overlapping with both low-grain and forage-free groups. This diet was positively associated with total bacterial abundance, mcrA (methanogens), and archaeal populations, reflecting a distinct microbial and fermentative profile compared with the other diets.

## 4 Discussion

### 4.1 Ruminal fermentation profile and acidosis risk

Considering that this study evaluated strategies for forage-free diets, it is essential to first discuss the inherent risk of such diets in predisposing animals to ruminal acidosis, thereby contextualizing our findings. Ruminal acidosis is a disorder caused by the excessive intake of highly fermentable carbohydrates, such as grains, leading to accelerated production of organic acids in the rumen. Under normal conditions, these acids are absorbed by the ruminal wall or neutralized by salivary buffers, maintaining pH between 5.6 and 6.5. However, when acid production exceeds the rumen’s absorptive and buffering capacities, which depend on saliva stimulated by chewing and rumination, non-physiological accumulation of acids occurs, progressively lowering pH. As ruminal pH drops below 5.6, acidosis sets in, compromising the ruminal microbiota, motility, and absorptive function. Moreover, at pH levels approaching 5.0, lactate concentrations increase suddenly because *Streptococcus bovis* shifts glucose fermentation toward lactate rather than SCFA. Since lactate is a stronger acid than SCFA (pKa 3.9 vs. 4.8), it accumulates further, and lactate-utilizing bacteria are inhibited below pH 5.0, preventing conversion of lactate into SCFA and exacerbating the pH decline (Castillo et al., 2004; Hernández et al., 2014; Krause & Oetzel, 2006; Nagaraja & Lechtenberg, 2007; Nagaraja & Titgemeyer, 2007).

Ruminal acidosis can be classified as acute or subacute. The acute form is characterized by pH < 5.0 and lactate accumulation (50–120 mmol/L) (Nagaraja & Lechtenberg, 2007). Our results indicate that animals did not develop acute acidosis, since ruminal pH averaged around 5.6 (Table 2) and lactate concentrations were approximately 14 mmol/L. However, the elevated lactate and SCFA concentrations point to signs of subacute acidosis (SARA). SARA typically occurs with pH values between 5.0 and 5.5, SCFA 150–225 mmol/L, lactate 0–5 mmol/L, and absence of overt clinical signs, although reductions in feed intake and subtle behavioral changes may be observed (Nagaraja & Lechtenberg, 2007).

Despite the significant reduction in ruminal pH in the forage-free diet (Table 2), the mean values (5.6) were above the diagnostic threshold for SARA. Nonetheless, the lactate concentration (13.96 mmol/L) suggests abnormal accumulation, exceeding typical subacute levels (0–5 mmol/L), alongside increased SCFA concentrations (155.2 mmol/L) and a reduction in the abundance of ciliate protozoa and fiber-degrading bacteria such as Fibrobacter succinogenes and Ruminococcus spp. However, these effects should be interpreted with caution, since the animals remained in a long-term confinement (147 days), a condition that naturally favors a decline in fibrolytic microbes due to continuous exposure to forage-free diet.

Another important factor is DMI reduction, often the most reliable indicator of SARA (Nagaraja & Lechtenberg, 2007). Notably, the forage-free diet promoted increased DMI and, consequently, greater FBW (Table 2). Thus, the results indicate an acidified ruminal environment with no clear evidence of classical SARA, indicating a possible microbiological adaptation to highly fermentable diets. Supporting this interpretation, Lopes et al. (2023) reported that replacing conventional chemical additives (monensin + virginiamycin) with Factor LM+ increased ADG, fecal pH, ruminal pH, and acetic acid production in confined cattle, confirming the additive’s potential to stabilize ruminal fermentation. Mechanistically, Factor LM+ may have supported ruminal pH control and microbial adaptation by altering membrane permeability and ion gradients in Gram-positive, acidogenic and lactate-producing bacteria, thereby limiting lactate accumulation and excessive ruminal acidification under forage-free conditions (Benchaar et al., 2008; Calsamiglia et al., 2007; Dorman & Deans, 2000; Nagaraja & Lechtenberg, 2007). However, the absence of repeated ruminal pH measurements throughout the days limits a definitive assessment of the occurrence of SARA under these conditions. Further studies with more frequent ruminal pH measurements, SCFA and detailed microbiota assessments would help to clarify the additive’s effects more precisely.

### 4.2 Nutrient intake and animal performance

Dry matter intake in ruminants is regulated by both physical and chemostatic mechanisms, as described by Mertens (1997), who also reported that dairy-type animals (such as those used in this study) have an intake capacity of approximately 1.3 % of their body weight in NDF. Additionally, diets with high grain content promote intense SCFA production, mainly acetate and propionate, which, once absorbed into the bloodstream and reaching the liver, can exceed metabolic capacity, triggering satiety signals before the rumen is physically full (Filho et al., 2023).

In the low-grain group (Table 2), NDF intake reached 1.5 % of BW, indicating regulation by ruminal fill, as expected under fiber-limiting conditions (Mertens, 1997). In contrast, high-grain and forage-free diets increased DMI due to greater energy density (as indicated by TDNI values in Table 2), suggesting chemostatic regulation. This is consistent with higher SCFA and propionate concentrations observed *in vivo* (Table 2) and *in vitro* (Table 4). Elevated SCFA concentrations resulted from diets with higher TDNI, especially in the forage-free group, which also showed significantly greater IVDOM (Table 4). Consequently, without a physical limitation, animals in the high-grain and forage-free groups could increase meal frequency, raising daily DMI and TDNI intake.

This led to greater fermentative efficiency, despite reductions in the abundance of the microbial groups quantified by qPCR (Table 3), ultimately resulting in higher FBW. Therefore, despite the reduction in ruminal pH described in the previous section, animals fed the forage-free diet did not show compromised performance, indicating that the plant-based metabolite additive effectively maintained rumen fermentation at healthy levels without impairing animal health or performance.

### 4.3 CH_4_ emissions and microbial adaptations

Enteric CH_4_ is a by-product of microbial degradation of carbohydrates, yielding SCFA (mainly acetate, propionate, and butyrate), CO_2_, H_2_, and microbial biomass. The accumulation of H_2_ in the rumen negatively affects fermentation rates and the growth of certain microbial populations; therefore, the activity of methanogenic archaea is essential for maintaining normal ruminal conditions by capturing H_2_ and converting CO_2_ into CH_4_ and H_2_O. While acetate and butyrate pathways release H_2_ and CO_2_, propionate pathway acts as a hydrogen sink, thereby reducing CH_4_ potential (Abbasi et al., 2018; Amanzougarene & Fondevila, 2020; Bhatta et al., 2009; Blümmel et al., 2005; Goopy et al., 2016; Morgavi et al., 2010). Therefore, strategies to reduce CH_4_ production include modulating the ruminal microbiota to shift SCFA production toward a higher proportion of propionate, which competes with methanogenic archaea for H_2_, or using additives that inhibit methanogens.

We observed lower estimated CH_4_ emissions *in vivo* for animals fed the forage-free diet (Table 2), whereas the *in vitro* assays indicated higher CH_4_ per unit of DM or DOM and a greater relative partitioning of degraded OM toward CH_4_ for this same diet (Table 4; Fig. 1). This apparent contradiction can be explained by the fundamental differences between both approaches. Under *in vivo* conditions, high-grain diets typically reduce ruminal pH, increase propionate formation, suppress fibrolytic activity, and limit H_2_ availability for methanogens, resulting in lower enteric CH_4_ emissions. In contrast, *in vitro* systems are highly buffered, preventing the natural pH decline and often promoting a more acetogenic fermentation pattern than would occur in the animal (Yánez-Ruiz et al., 2016). Additionally, the absence of digesta turnover, the accumulation of fermentation end-products, fine substrate grinding, headspace gas composition, and alterations in the microbial community during inoculum preparation create an artificial environment that frequently overestimates CH_4_ production from concentrate-rich substrates (Yánez-Ruiz et al., 2016). When animal performance is considered, the interpretation becomes clearer. Methane emissions expressed in g day^-1^ and per unit of DMI decreased from the low-grain to the forage-free diet (Table 2), indicating greater energetic efficiency and lower CH_4_ yield under forage-free diet. However, these findings should be interpreted with caution, *in vivo* emissions were estimated using predictive equations (Lingen et al., 2019). Direct measurements using respiration chambers or SF_6_ tracer techniques (Abdalla et al., 2012) would be necessary to further elucidate these relationships.

Another important factor for CH_4_ emissions is the diversity of the ruminal microbiota. Except for methanogenic microorganisms, the other microbes evaluated in this study are linked to fiber degradation (Table 3). The main fibrolytic bacteria include the Gram-negative *Fibrobacter succinogenes* and two Gram-positive species, *Ruminococcus albus* and *Ruminococcus flavefaciens*. Although *Prevotella* species are not highly cellulolytic, they produce xylanases and oligosaccharide-degrading enzymes, enabling them to utilize diverse polysaccharides and contribute significantly to xylan degradation as second-line degraders. Additionally, anaerobic fungi are important for fiber digestion, as they colonize plant tissues and appear capable of degrading lignified material resistant to other microbes. There is also growing evidence that ruminal protozoa may contribute to fiber digestion, although this capacity is not yet well understood (Krause et al., 2003; Tajima et al., 2001; Thoetkiattikul et al., 2013; Vasta et al., 2019).

Grain-rich diets favor amylolytic bacteria (up to 95 % of cultivable bacteria), due to their rapid growth rate and ability to ferment starch and soluble sugars more quickly than fibrolytic bacteria can degrade fiber. High-grain or forage-free diets also have variable effects on ruminal protozoa, including the complete elimination of protozoa in some animals, since protozoal populations cannot survive prolonged exposure to pH levels below 5.5. Furthermore, the production of lactic acid and the consequent reduction in ruminal pH inhibit the growth of most bacteria, especially fibrolytic species (Krause & Oetzel, 2006; Nagaraja & Titgemeyer, 2007; Nussio et al., 2006). In this study, we also observed a considerable reduction in Protozoa, Fungi, *Fibrobacter succinogenes, Ruminococcus albus, Ruminococcus flavefaciens*, and *Prevotella ruminicolla* (all involved in fiber degradation) in forage-free diet. Therefore, the lack or low inclusion of fiber in the high-grain and forage-free diets, along with the lower ruminal pH, reduced the abundance of these microorganisms, shifting the microbiota profile from cellulolytic to amylolytic and lactate-producing dominance.

### 4.4 Integrative analysis and practical implications

The PCA confirmed clear clustering of diets, integrating fermentation parameters, qPCR-quantified microbial groups, and animal performance data (Fig. 2). Low-grain diets clustered with higher fibrolytic bacteria, protozoa, undegraded OM, and higher A:P ratios, which are indicative of a fermentation profile dominated by fiber degradation due to the high forage content, which constituted 90 % of this diet (Table 1). While this supports ruminal stability, it was associated with lower feed efficiency due to reduced organic matter degradability and lower SCFA production (Fig. 1 and Table 4), reflecting limited energy availability from the diet and resulting in reduced FBW (Table 2).

High-grain diets showed an intermediate profile, partially overlapping with both low- and forage-free groups. These animals exhibited greater fermentative efficiency, evidenced by higher concentrations of SCFA and increased TDNI (Table 2), while still maintaining some fibrolytic populations (Table 3). This suggests that moderate grain inclusion can enhance energy supply and improve performance without completely suppressing the microbial fibrolytic populations evaluated by qPCR (Table 3).

Forage-free diets formed a distinct cluster, linked to higher SCFA, *in vitro* GP, IVDOM, DMI, microbial biomass partitioning, and FBW. These variables indicate a fermentation profile characterized by higher organic matter degradability and SCFA production, which supported superior growth performance. Although reductions in ruminal pH and fibrolytic bacteria were observed, these were expected adaptations to forage-free diets and did not compromise performance, as evidenced by the greater FBW (Table 2). The data suggest that the plant-based additive successfully modulated ruminal fermentation, enabling animals to cope with the acidotic risk inherent to forage-free diets while maintaining intake and performance.

Practically, these findings highlight that forage-free diets, when combined with effective rumen-modulating additives, can shorten finishing periods, improve productivity, and reduce CH_4_ emissions per kg BW gain. Nonetheless, careful management is required to ensure gradual adaptation, monitor ruminal pH, and maintain ruminal stability and microbial function to minimizes digestive disorders. Therefore, forage-free feeding with plant-derived additives emerges as a viable strategy for high-efficiency environmentally sustainable beef production.

## 5 Conclusion

The forage-free diet supplemented with a plant-based rumen-modulating additive enhanced DMI, fermentation efficiency, and FBW without compromising ruminal health, despite the expected reductions in pH and changes in the abundance of fibrolytic microorganism populations quantified by qPCR. The increased IVDOM, greater SCFA concentrations, and lower A:P ratio were consistent with enhanced energy availability to support superior animal performance. Reductions in CH_4_ emissions were estimated from *in vivo* data, indicating a lower CH_4_ intensity associated with improved productivity, which should be confirmed through direct, individual animal measurements using gold-standard techniques. Overall, these findings suggest that plant-derived additives may help stabilize ruminal fermentation in forage-free diets, enabling their safe application as a sustainable approach to achieve both high-efficiency animal performance and climate-smart livestock systems.

## Data availability statement

The data supporting the findings of this study are available from the corresponding author upon request.

## Ethical statement

**COMPROVANTE**

Comprovamos o recebimento da solicitação de uso de animais para o projeto intitulado "Produção de metano, consumo, desempenho, digestibilidade e qualidade de carne e carcaça de bovinos zebuínos e cruzados recriados em sistema de Integração Lavoura-Pecuária e terminados em confinamento Produção de metano, consumo, desempenho, digestibilidade e qualidade de carne e carcaça de bovinos zebuínos e cruzados recriados em sistema de Integração Lavoura-Pecuária e terminados em confinamento", protocolo do CEUA: 16/2018 sob a responsabilidade de Leandro Samia Lopes que envolve a produção, manutenção e/ou utilização de animais pertencentes ao filo Chordata, subfilo Vertebrata (exceto o homem) para fins de pesquisa científica (ou ensino).

## CRediT authorship contribution statement

**Vagner Ovani:** Writing – original draft, Data curation, Conceptualization. **Leandro Sâmia Lopes:** Supervision, Resources, Funding acquisition, Conceptualization. **Lumena Souza Takahashi:** Writing – review & editing, Investigation. **Patricia Spoto Corrêa:** Writing – review & editing, Investigation. **Patricia Righeto:** Writing – review & editing, Investigation. **Theyson Duarte Maranhão:** Writing – review & editing, Investigation. **Thaynã Gonçalves Timm:** Writing – review & editing, Investigation. **Helder Louvandini:** Supervision, Resources, Funding acquisition, Conceptualization. **Adibe Luiz Abdalla:** Supervision, Resources, Funding acquisition, Conceptualization.

## Declaration of competing interest

The authors declare that they have no known competing financial interests or personal relationships that could have appeared to influence the work reported in this paper.
